# A Comparative Study of Vacuum-Freeze-Dried and Hot-Air-Dried Gannan Navel Orange Slices: Physical Characteristics, Volatile/Non-Volatile Compounds, Antioxidant Activity, and Sensory Attributes

**DOI:** 10.3390/foods14244327

**Published:** 2025-12-15

**Authors:** Yan Liang, Qingna Wu, Qin Xiong, Jun Zhang

**Affiliations:** National Engineering Research Center of Navel Orange, Gannan Normal University, Ganzhou 341000, China

**Keywords:** gannan navel orange, dried orange slices, freeze-drying, hot-air drying, antioxidant activity

## Abstract

The Gannan navel orange (GNO) industry is large but constrained by limited deep-processing. This study employed vacuum freeze-drying (FD) and hot-air drying (HD) methods to prepare dried GNO slices, comparing their physical properties, volatile/non-volatile compounds, antioxidant activity, and sensory quality. Compared with the HD sample (Δ*E*= 10.11), the color changes were more pronounced in the FD sample (Δ*E*= 34.39), which appeared whiter and brighter. The FD method preserved more vitamin C (9.09% loss) than the HD method (27.28% loss). In contrast, HD samples contained significantly higher levels of total flavonoids, total phenols, hesperidin, narirutin and didymin, with respective increment percentages of 13.81%, 19.27%, 17.03%, 27.56% and 33.33% compared to FD samples. Volatile analysis revealed that drying treatments led to a decrease in terpene content compared with fresh slices (fresh 48.84% vs. FD 47.81% vs. HD 47.42%), while ester content increased (fresh 13.87% vs. FD 14.59% vs. HD 14.45%). Both methods reduced key monoterpenes (e.g., β-terpineol, terpinolene, 3-carene, α-terpineol, β-thujene and α-terpinene), possibly converting them into compounds such as p-mentha-1(7),8-diene-ol and thymol. Notably, HD samples contained much higher levels of harmful compounds 5-hydroxymethylfurfural and furfural. FD samples exhibited superior antioxidant activity and were preferred in sensory evaluation for appearance, aroma, texture, and taste. The findings indicate that FD offers advantages in terms of morphological retention, vitamin C preservation, antioxidant activity, safety and sensory perception, underscoring the market potential of FD navel orange slices as a high-value, healthy food product.

## 1. Introduction

The Gannan navel orange (GNO) is a geographical indication product from the Gannan region of Jiangxi Province, China. It is represented primarily by the Newhall navel orange cultivar (*Citrus sinensis* Osbeck cv. Newhall) [1]. Newhall navel oranges are plump and symmetrical with vibrant orange-red peels and crisp, tender, juicy pulp that has a balanced sweet and sour flavor. Previous research indicates that GNO are not only rich in nutrients such as vitamin C, B vitamins, organic acids, minerals, and dietary fiber, but also contain various bioactive compounds including flavonoids, phenolic acids, carotenoids, and essential oils [2,3,4]. As approximately 90% of GNO are sold as fresh fruit, oversupply is likely during the peak marketing period, leading to unsold fruit and diminished economic returns [4,5,6]. To enhance the comprehensive utilization value of GNO, alleviate pressure from the concentrated fresh fruit market, and mitigate losses for growers, researchers have conducted a series of studies on deep-processing technologies [7,8]. However, the variety of processed products currently developed remains limited, primarily focusing on navel orange jam, fruit cakes, juice, wine, and pies, and these products face issues such as low value-added and relatively constrained market demand. Therefore, there is an urgent need to develop new, nutritious and health-oriented deep-processed products from GNO.

Dried fruit slices represent a significant product category within the fruit and vegetable processing sector, referring to low-moisture food items produced by dehydrating fresh fruits after pre-treatments such as washing, peeling, and slicing [9]. This product offers advantages including ease of storage and transportation, extended supply periods, and the provision of unique flavors and textures. Current mainstream market products primarily include dried apples, banana chips, dried mangoes, and dried kiwifruit, with their consumer demand exhibiting continuous growth [10,11]. Regarding drying technology applications, HD remains the dominant industrial method due to its advantages of simple equipment and low cost. HD removes moisture by transferring heat from a drying medium to the material, driving water evaporation from the surface and from the interior through diffusion [12]. Although HD is simple and low-cost, high temperatures often cause shrinkage, color loss, and degradation of heat-sensitive components [12,13]. In contrast, FD process of fruits and vegetables involves freezing the material below its eutectic point and then removing ice by sublimation under vacuum, which maximally preserves the original morphology of the fruit and the content of bioactive substances like vitamins and polyphenols, endowing the product with a porous structure and excellent rehydration capacity [14,15]. Additionally, drying technologies including microwave drying, infrared drying, and their combination drying techniques are also under exploration, aiming to balance drying efficiency, product quality, and production costs [16]. However, these methods may still face challenges such as uneven heating, potential degradation of heat-sensitive nutrients, or compromised structural integrity, which could result in inferior product quality compared to FD.

To the best of our knowledge, only two previous studies have specifically addressed the drying of GNO slices [17,18]. The first employed ultrasound-assisted vacuum osmotic dehydration to prepare slices with the peel retained [17]. The second, published in Chinese, mainly investigated the influence of drying methods on non-enzymatic browning, also using unpeeled slices [18]. Notably, both studies kept the orange peel, which contains bitter compounds and other substances that may adversely affect the sensory quality and taste of the final product. In light of this, the present study takes a different approach by drying peeled GNO slices, with the aim of developing a dried product characterized by improved palatability and better-preserved nutritional components. This was achieved by comparatively analyzing the effects of two drying methods (HD and FD) suitable for industrial production on the physical characteristics (color, appearance), non-volatile components including vitamin C (VC), titratable acidity (TA), total phenolic content (TPC), total flavonoid content (TFC) and total soluble sugar content (TSS), volatile compounds, and antioxidant activity, as well as the sensory properties of dried navel orange slices without peel. The results of the present study will lay the foundation for the industrial production of high-quality dried GNO slices. The relevant research results could not only alleviate the pressure from the concentrated marketing of fresh fruits, reduce postharvest losses, and extend the industry chain, but also meet the increasing consumer demand for convenient, nutritious, natural, and healthy snack foods.

## 2. Materials and Methods

### 2.1. Chemical Reagents

Methanol, N,N-dimethylformamide (DMF), absolute ethanol, aluminum chloride hexahydrate, sodium carbonate, sodium nitrite, sodium hydroxide, and ascorbic acid (analytical grade) were purchased from XiLong Scientific Co., Ltd. (Shantou, China). D-Fructose, sodium dihydrogen phosphate dihydrate, potassium dihydrogen phosphate (anhydrous), disodium hydrogen phosphate dodecahydrate, gallic acid, and ferric chloride (analytical grade) were purchased from Sinopharm Chemical Reagent Co., Ltd. (Beijing, China). DNS reagent, 2,4,6-Tris(2-pyridyl)-s-triazine (TPTZ), 2,2-diphenyl-1-picryhydrazyl (DPPH, 97%), 2,2′-azino-bis(3-ethylbenzothiazoline-6-sulfonic acid) diammonium salt (ABTS, 98%), and Folin–Ciocalteu reagent (1 M) were purchased from Solarbio Science & Technology Co., Ltd. (Beijing, China). Acetonitrile (HPLC grade) was purchased from Anaqua Chemicals Supply (Houston, TX, USA). Deionized water for chromatography was prepared using a Milli-Q Gradient A10 system (Millipore, Billerica, MA, USA).

### 2.2. Preparation of Dried Orange Slices

Mature Newhall navel oranges (*Citrus sinensis* Osbeck cv. Newhall), which are commercially available, were purchased in Xinfeng County, Jiangxi Province, China. For this study, oranges that were free from mechanical damage or disease spots and were uniform in size (with a diameter of about 80 mm) were selected. Before the experiment, fruits were rinsed with running water to remove surface impurities and dried naturally at room temperature. The peel was subsequently removed using a professional peeling machine (Rimei 5384, Guangdong Jinda Hardware Products Co., Ltd., Foshan, China). Standardized slicing (thickness 5 ± 0.5 mm) was then performed using a precision slicer (QH-139, Foshan Qianghong Hotel Supplies Co., Ltd., Foshan, China). The experiment employed a completely randomized design with three biological replicates per treatment. Over ten fruits were selected for each replicate. After slicing, samples were thoroughly mixed and equally divided into three groups: HD group, FD group, and fresh group. Slices for the fresh group were immediately transferred to a −80 °C ultra-low temperature freezer for storage. Samples for the drying groups were subjected to the corresponding drying procedures immediately.

HD treatment: Orange slices were evenly placed in a preheated forced-air drying oven (BPG-9140A, Shanghai Yiheng Technology Co., Ltd., Shanghai, China) and dried at a constant temperature of 60 °C. This temperature was selected based on the preliminary experimental results. In preliminary experiments, four temperatures (40 °C, 50 °C, 60 °C, and 70 °C) were evaluated. At 40 °C and 50 °C, drying time was prolonged (>24 h) and the final product exhibited higher moisture content. At 70 °C, significant browning was observed, adversely affecting color quality. Therefore, 60 °C was selected as the optimal temperature for HD, balancing efficient drying time, low moisture content, and minimal browning. Samples were weighed hourly to monitor mass changes. Drying was considered complete when the mass difference between two consecutive weighings was less than 0.1%. The dried samples were immediately sealed and stored at −80 °C. The experiment was repeated three times, with each trial containing 90 orange slices.

FD treatment: Orange slices were pre-frozen at −80 °C for 6 h and then transferred to a vacuum freeze dryer (FD8-6 P, SIM International Group, Newark, NJ, USA) at vacuum pressure < 10 Pa and condenser temperature −80 °C for drying. Samples were weighed every 6 h during the initial phase (0–18 h) to monitor dehydration, and every 12–18 h thereafter to avoid nighttime operations. In both cases, samples were temporarily removed from the vacuum system for weighing. The drying endpoint was defined when the mass difference between two consecutive measurements was less than 0.1%. The final samples were then sealed and stored at −80 °C. The experiment was repeated three times, with each trial containing 90 orange slices.

### 2.3. Determination of Dehydration Curves, Moisture Content, and Water Activity

The fresh navel orange slices were weighed, and their weights were recorded. For the HD operation, samples were weighed every hour until the slices reached constant weight. For the FD operation, samples were weighed every 6 h during the initial phase (0–18 h), and every 12–18 h thereafter to avoid nighttime operations until the slices reached constant weight. The moisture ratio (MR) of the slices was calculated based on Equation (1). The final moisture content (MC1) of the dry slices and the initial moisture content (MC2) of the fresh slices were determined gravimetrically using Equations (2) and (3), respectively. This was achieved by drying the samples at 105 °C until they reached a constant weight [19]. The dehydration curves were plotted with the moisture ratio as the ordinate and the drying time as the abscissa. The water activity (a_w_) of the samples was measured at a temperature of 25 °C using a Novasina a_w_ Sprint TH-500 instrument (Pfäffikon, Switzerland).

(1) $$MR=\frac{Mt-Me}{M0-Me}\times 100\%$$

(2) $$MC1=\frac{Me-Md}{Me}\times 100\%\;$$

(3) $$MC2=\frac{M0-Md}{M0}\times 100\%$$

*Mt* is the weight of the slice at drying time ‘*t*’, *Me* is the constant weight after drying by HD or FD methods, *M*0 is the initial weight of the fresh slice, and *Md* is the weight of the sample after drying at 105 °C until it reaches a constant mass.

### 2.4. Color Measurement

A portable colorimeter (NR200, Shenzhen 3nh Technology Co., Ltd., Guangzhou, China) was used to measure the color of the navel orange slices at the flesh position, recording the *L*, *a*, and *b* values from the instrument. The *L* value indicates the lightness of the surface color; a higher *L* value indicates a whiter color. The *a* value represents the range from red to green; a larger positive value indicates a redder color, while a smaller negative value indicates a greener color. The *b* value represents the range from yellow to blue; a larger positive value indicates a yellower color, while a smaller negative value indicates a bluer color. The Δ*E* value represents the total color difference between two samples; a larger Δ*E* value indicates a greater color difference before and after drying. The calculation formula (Equation (4)) is as follows:

(4) $$\Delta E=\sqrt{{\left(\;L_{1}\;-L_{0}\;\right)}^{2}+\;{\left(\;a_{1}\;-a_{0}\;\right)}^{2}\;+\;{\left(\;b_{1}\;-b_{0}\;\right)}^{2}}$$

*L*_1_, *a*_1_, *b*_1_ are the measured values after drying; *L*_0_, *a*_0_, *b*_0_ are the measured values for the fresh sample.

### 2.5. Microscopy and Scanning Electron Microscopy Analysis

The dried navel orange slices were placed in a clean Petri dish and observed on the stage of a stereomicroscope (SZ810, Aote Optical Instrument Co., Ltd., Guangzhou, China). Scanning electron microscopy was used to observe the microstructure of the dried navel orange slices, following the method described in reference to minor modifications [19]. The dried navel orange slices were taken and cut into small pieces approximately 1 cm^2^ using scissors. After being sputter-coated with gold, the samples were observed using a scanning electron microscope (Quanta 450, FEI Co, Hillsboro, OR, USA).

### 2.6. Determination of Vitamin C (VC)

The VC content was determined using the 2,6-dichlorophenolindophenol method according to previous literature [1], and the results were expressed as grams of VC per 100 g of sample (g/100 g).

### 2.7. Determination of Titratable Acid (TA)

The TA content was determined using an acid-base titration method, following a previously described procedure [1]. The results are expressed as grams of citric acid equivalent per 100 g of sample (g CA/100 g).

### 2.8. Determination of Total Soluble Sugar (TSS) Content

The TSS content was determined with the 3,5-dinitrosalicylic acid (DNS) reagent according to a previously described method [20]. The results are expressed as grams of glucose equivalents per 100 g of sample (g Glu/100 g).

### 2.9. Determination of Total Phenolic Content (TPC) and Total Flavonoid Content (TFC)

Fresh navel orange slices (10 g) or dried navel orange slices (1.5 g) were weighed. The slices were cut into small pieces approximately 1 cm^2^ using scissors. Ten milliliters of 10% DMF/MeOH (*w*/*v*) was added, and the mixture was allowed to stand and soak for 30 min, followed by ultrasonic extraction (ultrasonication temperature 35 °C, frequency 25 kHz, time 30 min). After ultrasonication, the mixture was centrifuged (centrifugation time 5 min, speed 5000 rpm). The supernatant was collected. The extraction was repeated three times, and the extracts were combined. The combined extract was finally diluted to 50 mL with 10% DMF/MeOH (*w*/*v*). This sample solution was stored at −80 °C for subsequent analysis of total phenolics, total flavonoids, antioxidant activity, and high-performance liquid chromatography (HPLC) quantification.

The TFC was determined according to Lai et al. [21] and expressed as grams of quercetin equivalent per 100 g of sample (g QE/100 g). Similarly, the TPC was measured following the method described by Hou et al. [3] and reported as grams of gallic acid equivalent per 100 g of sample (g GA/100 g).

### 2.10. HPLC Determination of Major Flavonoid Compounds

The HPLC analysis of three major flavonoid compounds, including narirutin, hesperidin, and didymin, was conducted in accordance with a previously established method, with minor adjustments [22]. The slice extract obtained in Section 2.9 was diluted twofold with 10% DMF/MeOH, and the diluted extract was filtered through a PTFE filter (0.22 μm) to obtain the sample for HPLC analysis. Analysis was performed using an XBridge™ C18 reversed-phase column (5 μm, 4.6 × 150 mm) with detection at 210 nm. The mobile phase consisted of acetonitrile (A) and ultrapure water (B) with the following gradient program: 0–40 min, 10–20% A; 40–45 min, 20–40% A; 45–60 min, 40–55% A; 60–65 min, 55–90% A; 65–70 min, 90% A; 70–80 min, 90–10% A. Three major flavonoids were identified in the orange slices by comparing their retention times and UV absorption spectra with those of standard compounds. Quantification was performed using external standard calibration curves: narirutin (y = 86,107x – 617,808, R^2^ = 1.00, linear range 62.5–500 μg/mL), hesperidin (y = 73,685x – 182,759, R^2^ = 1.00, linear range 15–240 μg/mL), and didymin (y = 103,818x + 791,514, R^2^ = 0.9999, linear range 125–1000 μg/mL). The respective retention times for these compounds were 26.72, 32.25 and 46.18 min.

### 2.11. Antioxidant Activity Assay

The ABTS and DPPH radical scavenging capacities and the FRAP antioxidant assay were determined according to the method of Long et al. [23]. Each test was performed in triplicate. The ABTS radical scavenging capacity was calculated using Equation (5):

(5) $$Scavenging\;rate\;of\;ABTS\left(\%\right)=[1-\frac{A1-A0}{A2}]\times 100\%$$

*A*1: Average absorbance of the solution containing ABTS and sample; *A*0: Average absorbance without ABTS solution; *A*2: Average absorbance without sample but with ABTS solution.

The DPPH radical scavenging capacity was calculated using Equation (6):

(6) $$Scavenging\;rate\;of\;DPPH\left(\%\right)=[1-\frac{A1-A0}{A2}]\times 100\%$$

*A*1: Average absorbance of the solution containing DPPH and the sample; *A*0: Average absorbance in the absence of DPPH solution; *A*2: Average absorbance in the absence of the sample but containing DPPH solution.

The ABTS and DPPH radical scavenging capacities, as well as the FRAP antioxidant activity, were all expressed as the vitamin C equivalent per 100 g of sample (g VC/100 g).

### 2.12. Headspace Gas Chromatography-Mass Spectrometry (HS-GS-MS) Analysis

Three grams of the sample were placed into a 20 mL headspace bottle and immediately sealed for analysis. The analysis was performed using an Agilent 8890B gas chromatograph coupled with a 7697A headspace sampler and a 7000D mass selective detector featuring an inert electron impact (EI) ion source. The ionization voltage was set to 70 eV. The analyte compounds were separated using a VF-WAXms capillary column (25 m × 0.25 mm × 0.2 µm) with a constant flow rate of 1 mL/min of 99.999% helium as the carrier gas. The headspace oven temperature was set to 130 °C, the loop temperature to 150 °C, the transfer line temperature to 170 °C, the vial equilibration time to 20 min, and the GC cycle time to 35 min. The GC oven temperature program was as follows: hold at 40 °C for 2 min, then ramp to 100 °C at 5 °C/min, followed by a ramp to 230 °C at 15 °C/min, and hold for 5 min. Samples were injected in split mode (10:1 split ratio) with an injection volume of 1 µL and an inlet temperature of 180 °C. The ion source temperature was 230 °C and the quadrupole temperature was 150 °C. Full scan mode was used for mass spectral acquisition with an m/z range of 30–1000 and a scan rate of 3.2 scans/s. To evaluate analytical system stability during the run sequence, a quality control (QC) sample was prepared. After mass spectrometry detection, the raw GC/MS data were pre-processed using the MassHunter Workstation Quantitative Analysis software (v10.0.707.0), with the quantification based on internal standards. Metabolites were identified using the Fiehn and NIST public databases, with a NIST similarity of at least 80%. To reduce errors from sample preparation and instrument instability, the peak response intensity was normalized using the sum normalization method to obtain the normalized data matrix. Variables exhibiting a relative standard deviation (RSD) > 30% in QC samples were removed.

### 2.13. Sensory Evaluation

The sensory evaluation was conducted according to the method described by Mahjoorian et al. [24]. The Experimental Animal Welfare and Ethics Committee of Gannan Normal University confirmed that this project constituted a minimum-risk study and granted an exemption from ethical review. A total of twenty panelists (ten females and ten males) aged 20 to 25 were invited. All the panelists performed the same task over ten times and received sufficient training for the evaluation. Informed consent for participation was obtained from all subjects involved in the study. The samples were placed on disposable trays that were labeled with three random three-digit codes. The samples were presented in a random order. The evaluation was performed in a sensory analysis room maintained at a temperature between 20 and 22 °C. During the evaluation, the panelists were seated separately in booths and were prohibited from communicating verbally to minimize distractions and interactions. Before the sensory analysis began, the panelists were given instructions on how to proceed during the test. Panelists evaluated the color, appearance, odor, texture, crispness, and taste of navel orange slices subjected to different drying methods using a 9-point hedonic scale (1 = dislike extremely, 3 = dislike, 5 = neither like nor dislike, 7 = like, 9 = like extremely). Comments from each panelist were recorded on a questionnaire. Each sensory panelist participated in three tests for each sample. The data were expressed as the mean score for each attribute and plotted on a radar chart.

### 2.14. Data Analysis

The experimental data were processed and analyzed using Origin 2017, SPSS 25.0, Microsoft Excel 2010, and GraphPad Prism 9.5. The R package ropls (version 1.6.2) was used for analyzing HS-GC-MS data through principal component analysis (PCA), correlation analysis, and orthogonal partial least squares discriminant analysis (OPLS-DA). Additionally, Student’s *t*-tests and fold-change analyses were performed. Statistical differences were analyzed using one-way analysis of variance (ANOVA), followed by Tukey’s post hoc test at a *p*-value of less than 0.05. For the HS-GC-MS data, significantly different metabolites were identified based on variable importance in projection (VIP) values from the OPLS-DA model and *p*-values from Student’s *t*-tests. Metabolites with VIP > 1 and *p*-value < 0.05 were classified as significantly different.

## 3. Results

### 3.1. Effects of Different Drying Methods on the Appearance and Color of Dried GNO Slices

**Figure 1:**
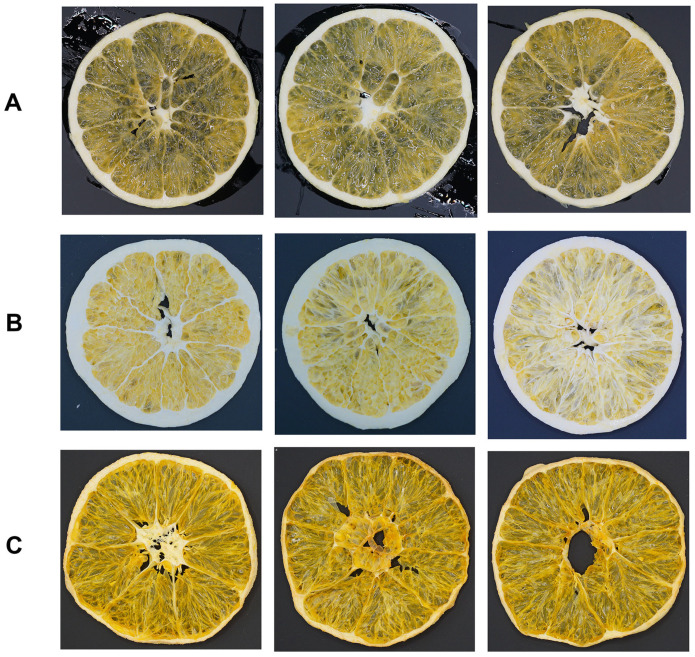
(**A**) Fresh navel orange slices; (**B**) Dried navel orange slices obtained by FD; (**C**) Dried navel orange slices obtained by HD. FD: vacuum freeze-drying; HD: hot-air drying.

Figure 1 displays the apparent morphology of fresh navel orange slices and those subjected to two different drying methods. As shown in Figure 1A, fresh navel orange slices exhibit a typical orange-yellow color. Samples processed by FD (Figure 1B) showed a significantly lighter color, appearing pale yellowish-white, and largely retained their original slice structure and thickness. In contrast, HD samples (Figure 1C) exhibited a darker, brownish-yellow color and pronounced shrinkage deformation.

**Figure 2:**
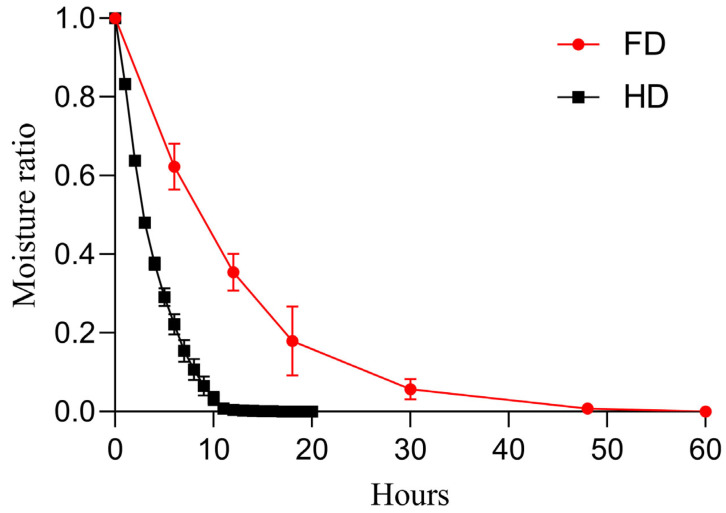
Drying curves showing the moisture ratio over time. FD: vacuum freeze-drying; HD: hot-air drying.

Figure 2 shows the curves of moisture ratio versus time during the drying process, clearly illustrating distinct dehydration characteristics between the two drying methods: the FD process exhibited a faster dehydration rate in the initial stage but slowed down later, requiring approximately 60 h to reach complete dryness; whereas the HD process showed a relatively consistent and stable moisture decline trend, with a shorter overall dehydration time of approximately 12 h to achieve complete dryness. The average initial moisture content of the fresh slices was 85.83 ± 0.11%. Additionally, no significant difference in final moisture content was observed between the HD- and FD-treated samples (*p* > 0.05), with respective values of 7.12 ± 1.05% and 6.84 ± 1.24%. These low moisture content levels suggest that both techniques effectively dehydrated the slices. Furthermore, the water activity (a_w_) was measured, with the results indicating that the FD samples (0.32 ± 0.02) had a significantly lower a_w_ than the HD samples (0.45 ± 0.01) (*p* < 0.05).

The data presented in Table 1 offer quantitative insights into the effects of drying treatments on the color of the samples. Regarding color parameters, FD samples had the highest *L* value (74.90 ± 0.61), which was significantly higher than the values for HD samples (49.21 ± 0.65) and fresh samples (47.54 ± 0.40) (*p* < 0.05). This indicates that the FD samples were the lightest and had the palest color. The *a* (4.36 ± 0.38) and *b* (30.47 ± 0.92) values of the FD samples were also significantly higher than those of the other two groups. This reflects changes in their red-green and yellow-blue color values, respectively. The total color difference (Δ*E*) value revealed that the color change in the FD samples (34.39 ± 0.35) was considerably greater than that of the HD samples (10.11 ± 0.19) (*p* < 0.05).

**Figure 3:**
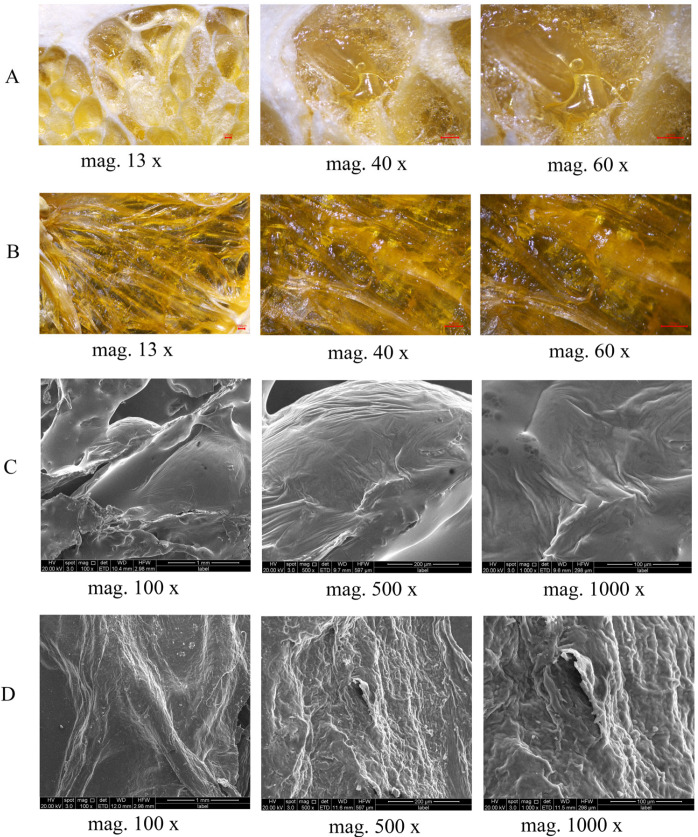
(**A**) Surface morphological structure of FD slices observed under optical microscope; (**B**) Surface morphological structure of HD slices observed under optical microscope; (**C**) Surface morphological structure of FD slices observed under scanning electron microscope; (**D**) Surface morphological structure of HD slices observed under scanning electron microscope. FD: vacuum freeze -drying; HD: hot-air drying.

Figure 3 reveals the effects of different drying methods on the microstructure of navel orange slices using optical microscopy (OM) and scanning electron microscopy (SEM). Results from Figure 3A (FD-OM) and Figure 3C (FD-SEM) are consistent, clearly showing that FD samples possess a relatively intact surface, low cellular wrinkling ratio, and no large-scale collapse. This structural characteristic explains the slight volume expansion observed in FD samples. In sharp contrast, Figure 3B (HD-OM) and Figure 3D (HD-SEM) reveal that HD samples possess a dense structure with severe shrinkage and collapse on the surface.

### 3.2. Effect of Different Drying Methods on Non-Volatile Components of GNO Slices

As shown in Table 2, to facilitate comparison, all data were expressed on a dry matter basis, taking into account the moisture content. Table 2 shows that the drying treatments had a significant effect on the content of the main functional components in navel orange slices. Fresh samples had the highest VC content (0.22 g/100 g); HD caused the greatest VC loss (0.16 g/100 g), and its content was significantly lower than that of fresh and FD samples (*p* < 0.05). FD samples had the highest TA content (1.51 g CA/100 g), significantly higher than fresh samples (1.23 g CA/100 g); HD samples (1.36 g CA/100 g) showed no significant difference compared to either. TFC and TPC showed similar trends: TFC (1.81 g QE/100 g) and TPC (0.83 g GA/100 g) in HD samples were significantly higher than in fresh samples (1.52 g QE/100 g, 0.70 g GA/100 g) and FD samples (1.56 g QE/100 g, 0.67 g GA/100 g). TSS content decreased significantly after drying, with fresh samples having the highest value (39.13 g Glu/100 g); there was no significant difference between FD and HD samples (32.35 g Glu/100 g and 28.56 g Glu/100 g, respectively) (*p* > 0.05). This indicates that hot-air drying has advantages in promoting the retention or transformation of flavonoids and phenolics but causes greater destruction of heat-sensitive VC.

**Figure 4:**
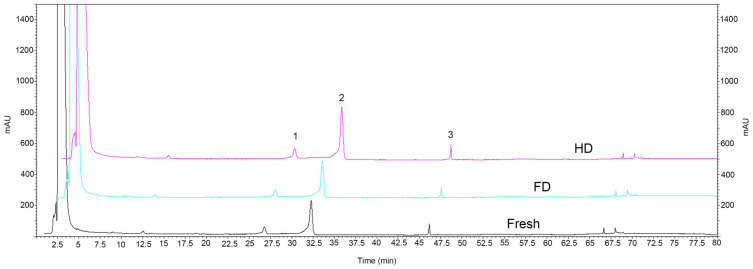
HPLC profile of navel orange slices detected at 210 nm wavelength. FD: vacuum freeze-drying; HD: hot-air drying. Peaks 1–3 are narirutin, hesperidin and didymin, respectively.

HPLC analysis revealed that navel orange slices mainly contained three flavonoids: narirutin, hesperidin, and didymin (Figure 4). The narirutin content was highest in the HD samples (0.47 g/100 g), which was significantly higher than in the FD samples (0.34 g/100 g) (Table 3). There was no significant difference between the HD and fresh samples (0.46 g/100 g) (*p* > 0.05). The hesperidin and didymin content was highest in the HD samples (1.82 g/100 g and 0.09 g/100 g, respectively) as well, which was significantly higher than in the fresh (1.56 g/100 g and 0.05 g/100 g) and FD (1.51 g/100 g and 0.06 g/100 g) samples; the contents of these two flavonoids in FD samples showed no significant difference compared to fresh samples (*p* > 0.05).

### 3.3. Effects of Different Drying Methods on Antioxidant Activity of GNO Slices

The results of the three different antioxidant assays (ABTS, DPPH and FRAP) are consistent and indicate that drying treatments significantly reduced the antioxidant capacity of navel orange slices (Table 3). Compared with fresh slices (all values expressed on a dry basis), samples subjected to either FD or HD exhibited significant decreases (*p* < 0.05) in ABTS, DPPH radical scavenging capacity, and FRAP ferric ion reducing power. Specifically, fresh samples showed the highest values among all groups for ABTS (0.70 g VC/100 g), DPPH (0.35 g VC/100 g), and FRAP (0.31 g VC/100 g). This suggests that the drying process inevitably led to the loss or degradation of antioxidant compounds in navel orange slices.

Further analysis revealed that FD treatment was significantly better than HD at preserving antioxidants. The ABTS value of FD-dried samples (0.62 g VC/100 g) was significantly higher than that of HD samples (0.57 g VC/100 g). For DPPH, FD samples (0.32 g VC/100 g) likewise demonstrated significantly higher values than HD samples (0.29 g VC/100 g). The same trend was observed for FRAP values, with FD samples (0.26 g VC/100 g) being significantly higher than HD samples (0.21 g VC/100 g) (*p* < 0.05). These results consistently indicate that the FD process more effectively preserves antioxidant components in navel orange slices.

### 3.4. Effect of Different Drying Methods on Volatile Components of GNO Slices

**Figure 5:**
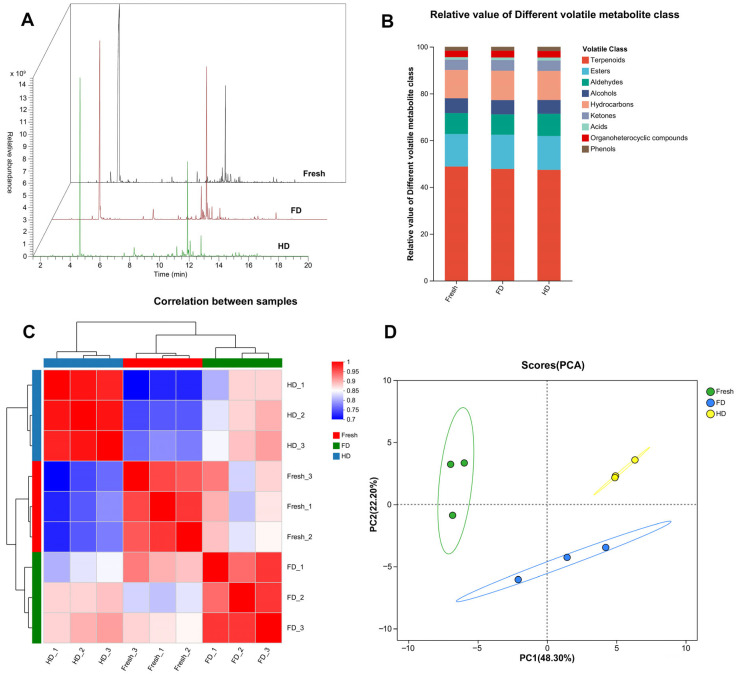
(**A**) Total ion chromatography; (**B**) Relative content of classified volatile compounds; (**C**) Heatmap illustrating the correlations of the samples; (**D**) PCA of the samples. FD: vacuum freeze-drying; HD: hot-air drying.

The effects of FD and HD methods on the volatile components of navel orange slices were analyzed using headspace-gas chromatography-mass spectrometry (HS-GC-MS). As shown in Figure 5A, the total ion chromatograms (TIC) intuitively display differences in chromatographic peaks of volatile compounds between FD- and HD-treated samples, indicating that the drying method significantly altered the composition and relative abundance of volatile substances. Figure 5B further compares the volatile compounds by category, showing that the main volatile substances in orange slices include terpenoids, esters, aldehydes, and alcohols. Furthermore, significant differences existed in the relative content of each compound class between FD and HD samples, particularly for key flavor compounds such as terpenoids, aldehydes, and alcohols. Specifically, the terpenoid content in fresh slices (48.84%) was higher than that in the FD (47.81%) and HD (47.42%) treatment groups. Conversely, the ester content in fresh slices (13.87%) was lower than that in the FD (14.59%) and HD (14.45%) groups. For HD samples, their aldehyde content (9.54%) was higher than that in fresh slices (8.97%) and FD products (8.74%); while their alcohol content (5.84%) was lower than that in fresh slices (6.27%) and FD products (6.06%). It is speculated that some alcohols were oxidized and converted into aldehydes during the HD heating process. The heatmap analysis in Figure 5C visually presents the correlation patterns among samples, showing higher correlations between samples subjected to the same drying treatment (e.g., among FD samples, among HD samples) and lower correlations between samples subjected to different drying treatments (FD vs. HD). Simultaneously, the correlation between FD and fresh slice samples was higher than that between HD and fresh slice samples, indicating that FD samples exhibited smaller differences in volatile components compared to fresh samples than HD samples did. This conclusion is strongly supported by the principal component analysis (PCA) results in Figure 5D: The PCA score plot shows clear spatial separation between FD and HD samples along the PC1 axis, with FD samples being spatially closer to fresh slice samples. This further confirms that the two drying processes led to significant differences in the overall volatile profile of dried navel orange slices, and the difference in volatiles between FD samples and fresh samples was smaller.

**Figure 6:**
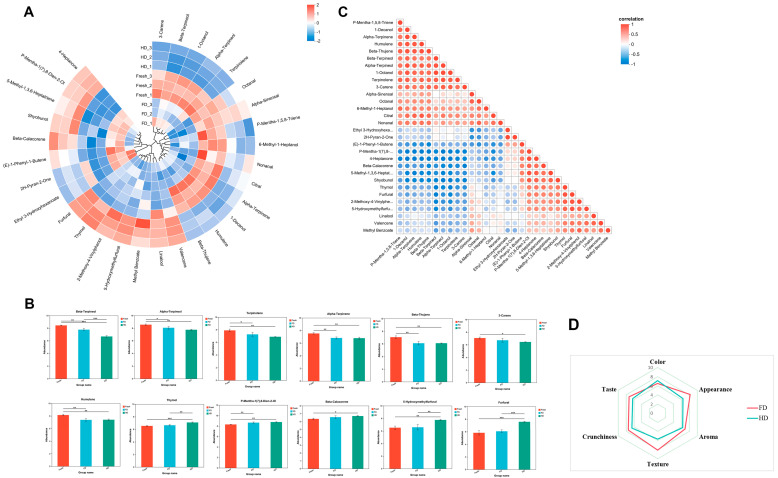
(**A**) Circular heatmap illustrating the relative content of differential volatile compounds; (**B**) Relative abundance of typical volatile compounds; (**C**) Triangular bubble heatmap illustrating the correlations between differential volatile compounds; (**D**) Radar chart illustrating the sensory evaluation results; FD: vacuum freeze-drying; HD: hot-air drying; *, ** and ***, indicate significant differences at the *p* < 0.05, 0.01 and 0.001 levels, respectively.

An in-depth analysis of differential volatile compounds is shown in Figure 6. The circular heatmap in Figure 6A details the relative content of differential volatile compounds between FD and HD samples. Regardless of whether freeze-drying or hot-air drying was used, the content of several major monoterpenes, including beta-terpineol, terpinolene, 3-carene, alpha-terpineol, beta-thujene, and alpha-terpinene, significantly decreased relative to fresh slices. Conversely, the relative content of monoterpenes such as p-mentha-1(7),8-dien-ol and thymol in dried slices was significantly higher than in fresh slices (Figure 6B). It is speculated that they may be converted from other monoterpene compounds during drying. Compared to freeze-dried products and fresh slices, the relative content of harmful substances 5-hydroxymethylfurfural (5-HMF) and furfural significantly increased in hot-air dried products, which may be related to the promotion of the Maillard reaction of monosaccharides during the heating process.

The triangular bubble heatmap in Figure 6C reveals the correlation network among these differential volatile compounds, with bubble size representing the strength of the correlation. Significant positive or negative correlations can be observed among several monoterpenes. The Pearson correlation coefficients between alpha-terpineol and p-mentha-1(7),8-dien-ol, and thymol were −0.9581 and −0.845, respectively; between beta-terpineol and p-mentha-1(7),8-dien-ol, and thymol were −0.8868 and −0.935, respectively; terpinolene, 3-carene, beta-thujene, and alpha-terpinene all showed significant negative correlations with p-mentha-1(7),8-dien-ol, with correlation coefficients of −0.89, −0.889, −0.949, and −0.946, respectively. Simultaneously, terpinolene and 3-carene both showed significant negative correlations with thymol, with correlation coefficients of −0.785 and −0.81, respectively. This suggests they may be closely linked in biosynthetic or degradation pathways.

### 3.5. Sensory Evaluation

As shown in the radar chart (Figure 6D), significant differences exist in the sensory quality of navel orange slices subjected to different drying methods. The results demonstrate that FD samples significantly outperformed HD samples across all sensory dimensions. Specifically, the FD group exhibited a substantially larger radar chart profile than the HD group, with all dimension scores positioned farther from the center point, visually confirming the superior sensory acceptability of freeze-dried products. Analysis of the radar plot morphology reveals that the FD curve is full and extends relatively uniformly toward all dimensions, indicating that freeze-drying comprehensively preserves multiple sensory attributes of navel orange slices. In contrast, the contracted profile of the HD group suggests diminished overall sensory scores due to hot air drying, with particularly poor performance in critical indicators such as crunchiness, texture, and appearance. However, FD products scored lower in color due to their pale color, which lacks the vibrant orange hue characteristic of fresh navel oranges.

## 4. Discussion

As a protected geographical indication product, the GNO was ranked first in China’s “Top Ten Regional Public Brands” and was included in the initial list of “China-EU 100 + 100” geographical indications products that have been granted mutual recognition [25]. However, constrained by the lack of efficient processing technologies, the GNO industry currently focuses primarily on fresh consumption, with deep-processed products accounting for a low proportion. To address this issue, this study systematically investigated the preparation technology for dried GNO slices, elucidating the differences in physical properties, non-volatile/volatile compounds, antioxidant activity, and taste between slices processed by FD and HD.

Regarding color, FD samples exhibited a light yellowish-white hue (Figure 1B). Their highest *L* value (74.90), *a* value (4.36), *b* value (30.47), and maximum total color difference Δ*E* (34.39) (Table 1) indicated significantly greater lightening compared to HD samples (Δ*E* = 10.11) and fresh samples. This pronounced color-bleaching effect possibly stemmed from pigment degradation (e.g., carotenoids) and alterations to light-scattering structures during FD, consistent with the significant fading reported in freeze-dried berries [15]. Previous research indicates that the porous structure formed by FD is the primary reason for enhanced light scattering and a lighter color [26,27]. Intense Mie scattering might occur within the dried FD tissue, resulting in the vast majority of visible light being reflected and the slice appearing opaque white or pale yellow. In contrast, the brownish-yellow color of HD samples (Figure 1C) and their moderate color difference conformed to the general pattern of browning induced by heat-driven Maillard reaction and caramelization, a common phenomenon in hot-air-dried fruits and vegetables [28,29].

The dehydration kinetics curve (Figure 2) clearly demonstrates the fundamental difference between the two drying modes: FD exhibits a higher initial dehydration rate during the rapid sublimation phase of ice crystals, which significantly slows down later due to increased mass transfer resistance, resulting in a total duration of up to 60 h. In contrast, HD maintains a relatively stable dehydration rate driven by continuous hot air convection, requiring only about 12 h. This result is highly consistent with the findings on the drying characteristics of apple slices [30,31], further supporting the theoretical framework that FD efficiency is limited by internal mass transfer within the material, while HD efficiency is governed by external convective conditions.

The results of this study indicate that the drying method significantly impacts the retention of major functional components in navel orange slices. Compared with fresh samples, HD demonstrated superiority in promoting the accumulation of flavonoids and total phenolics. Specifically, HD treatment not only significantly increased TFC and TPC but also yielded the highest levels of the three main monomeric flavonoids (narirutin, hesperidin, and didymin). This phenomenon aligns with previous research suggesting that moderate heat treatment may disrupt plant cell wall structures, facilitating the release of bound phenolics, or inactivate oxidase activity to reduce phenolic degradation [21,32,33]. It indicates that similar mechanisms may occur during HD, promoting the release or transformation of specific flavonoid components such as hesperidin. However, HD treatment caused severe damage to heat-sensitive components, resulting in the greatest loss of VC, and TSS content was also significantly reduced. This is consistent with the general pattern of VC oxidative decomposition and potential Maillard reaction consuming reducing sugars during thermal processing [34,35]. In comparison, FD treatment showed significant advantages in preserving heat-sensitive components; its VC content was significantly higher than HD and showed no significant difference from fresh samples. Concurrently, FD samples exhibited the highest TA content, and hesperidin and didymin levels were comparable to fresh samples, indicating that its low-temperature dehydration process effectively suppressed the degradation and transformation of related components.

The data presented in Table 3 of this study clearly demonstrate that drying treatments significantly reduced the antioxidant capacity of navel orange slices. Specifically, the ABTS, DPPH radical scavenging capacity, and FRAP ferric reducing antioxidant power of samples subjected to FD and HD were significantly lower than those of fresh samples (*p* < 0.05). This confirms that the drying process caused the loss or structural damage of natural antioxidant-active substances in navel oranges. This finding is consistent with numerous studies on fruit and vegetable drying, which generally attribute the degradation of core antioxidant components such as phenolics and VC during dehydration to thermal effects, oxidation reactions, and physical losses [13,36].

Further analysis revealed that the FD method was significantly superior to the HD method in preserving antioxidants in navel oranges. The ABTS, DPPH, and FRAP values of the FD samples (0.62, 0.32, and 0.26 g VC/100 g, respectively) were significantly higher than those of the corresponding HD samples (0.57, 0.29, and 0.21 g VC/100 g, respectively). This difference primarily stems from the temperature characteristics of the two processes: FD dehydrates via sublimation under deep freezing and vacuum conditions, and its low temperature minimizes thermal damage to thermolabile antioxidants such as vitamin C and phenolics. In contrast, HD relies on hot air at relatively high temperatures for dehydration. Prolonged thermal exposure readily induces degradation of thermosensitive components, accelerates residual oxidase activity (enzymatic browning), and may promote non-enzymatic browning processes like the Maillard reaction, leading to a significant decline in antioxidant activity [36].

This study systematically analyzed the effects of FD and HD on the volatile components of dried GNO slices using HS-GC-MS technology. The results demonstrate that the drying method significantly reshaped the composition and relative abundance of volatile substances in the slices. TICs and categorical comparisons (Figure 5A,B) visually revealed significant differences in key flavor compounds such as terpenes, esters, aldehydes, and alcohols between FD and HD samples. Compared with fresh slices, drying treatments generally led to a decrease in terpene content (fresh 48.84% vs. FD 47.81% vs. HD 47.42%), while ester content increased (fresh 13.87% vs. FD 14.59% vs. HD 14.45%). Notably, HD samples exhibited higher aldehyde content (9.54%) and lower alcohol content (5.84%), significantly differing from fresh slices (aldehyde 8.97%, alcohol 6.27%) and FD samples (aldehyde 8.74%, alcohol 6.06%). This aligns with the phenomenon observed in previous citrus drying studies, in which the conversion of alcohols to aldehydes was promoted by hot-air drying [37,38]. This suggests that heating during HD may facilitate the oxidation of alcohols. The correlation patterns between samples (Figure 5C) and PCA (Figure 5D) further confirmed that FD samples were closer to fresh slices in their overall volatile profile, showing higher correlation and spatial proximity; whereas FD and HD samples exhibited significant separation, highlighting the decisive influence of the drying process on product flavor.

In-depth analysis of the differential volatile compounds (Figure 6A,B) revealed that, regardless of FD or HD, the contents of several major monoterpenes, such as β-terpineol, terpinolene, 3-carene, α-terpineol, β-thujene, and α-terpinene, were significantly decreased compared to fresh slices. Conversely, the relative contents of monoterpenic alcohols like p-mentha-1(7),8-dien-9-ol and thymol were significantly increased in the dried samples, suggesting that the drying process might trigger the transformation of monoterpenes. Notably, significantly elevated levels of 5-hydroxymethylfurfural (5-HMF) and furfural were detected in HD samples. These findings are consistent with previous research on the formation of furanic compounds in thermally processed fruits [39,40]. They suggest that heating during HD promotes the Maillard reaction between reducing sugars and amino acids, as well as sugar degradation. Correlation network analysis (Figure 6C) revealed significant negative correlations among a series of monoterpene compounds (e.g., correlation coefficients between α-terpineol and p-mentha-1(7),8-dien-9-ol, thymol were −0.9581 and −0.845, respectively; coefficients between β-terpineol and the latter two were −0.8868 and −0.935, respectively). This strongly suggests close interconversion relationships within the biosynthetic or degradation pathways of these compounds.

Based on the differential patterns and strong correlation evidence above, this study proposes the following potential biotransformation pathway for monoterpenes during drying. Some decreased monoterpenes (e.g., β-terpineol, terpinolene, 3-carene, α-terpineol, β-thujene, α-terpinene) might be transformed, under drying conditions via reactions such as oxidation and hydration, into the increased monoterpenic alcohols (e.g., p-mentha-1(7),8-dien-9-ol, thymol) (see Figure S1 in the Supplementary File). HD involves higher temperatures than FD and likely accelerates oxidation and hydration processes. This explains the significantly higher thymol content observed in HD samples compared to FD samples (Figure 6B), which aligns with Chen et al.’s report on the temperature-promoted oxidative transformation of terpenes [41]. The proposal of this transformation pathway provides an important theoretical basis for a deeper understanding of the biochemical mechanisms by which different drying methods shape the characteristic flavor of dried GNO slices.

Sensory evaluation radar chart analysis in this study indicated that different drying methods significantly affected the quality of dried navel orange slices. The FD group scored significantly higher than the HD group in dimensions of crispness, texture, appearance, and taste. Its radar chart displayed a plump and uniform contour, reflecting that freeze-drying technology better preserved the product’s sensory characteristics. Conversely, the comprehensive inward contraction of the HD group across key indicators revealed quality deterioration caused by thermal effects. Notably, the FD product scored lower than the HD group in the color dimension. The sublimation drying mechanism of FD has been demonstrated to preserve the structural integrity of the cellular skeleton [42], thereby contributing to its distinctive crispness and texture. In contrast, high-temperature dehydration (HD) intensifies the Maillard reaction and causes the loss of volatile compounds [41], resulting in flavor deterioration and hardening of the texture. Notably, due to its longer operational time (~60 h for FD vs. ~12 h for HD), FD is more energy-intensive than HD. At an industrial scale, this leads to significantly higher operating costs, making the feasibility of FD highly dependent on local energy prices. In contrast, HD offers greater energy efficiency and lower production costs, rendering it a more economically viable option for many applications. Therefore, selecting a drying method in practice requires balancing target product quality with production costs. A comprehensive feasibility assessment should consider not only energy use, but also end-product value, market price, and required quality standards. Future research should focus on optimizing FD process parameters (e.g., pre-freezing rate, sublimation temperature, and vacuum degree) to mitigate color deterioration and enhance retention of total phenols and flavonoids. Additionally, exploring combined FD-HD strategies is warranted to balance product quality (color, morphology, nutrition, flavor, and safety) with processing efficiency and cost.

## 5. Conclusions

This study evaluated the effects of FD and HD on the quality of dried navel orange slices. The results revealed the distinct characteristics of each method. FD demonstrated superior retention of VC (20% higher), organic acids (9.93% higher), and antioxidant activity (8.06% higher in ABTS, 9.31% higher in DPPH, and 19.23% higher in FRAP) compared to HD, while preventing the formation of harmful thermal degradation by-products such as 5-HMF and furfural. However, FD resulted in poorer color retention and longer processing time. Compared with FD, HD was more effective in increasing the total flavonoid content, with particular increases seen in hesperidin (17.03%), narirutin (27.56%), and didymin (33.33%). These findings provide a scientific basis for selecting navel orange drying processes. HD is preferable if the primary objective is flavonoid enrichment, whereas FD should be prioritized if VC, acidity, texture, flavor integrity, and antioxidant activity must be preserved concurrently. The study also found that the drying method influences the transformation network of monoterpene compounds, indicating a direction for regulating volatile flavor quality. The GNO slices obtained by FD method in this study offer notable advantages, including excellent shape retention, high VC preservation, strong antioxidant activity, reliable safety, and desirable texture. These qualities position them as a promising natural health product with significant market potential.

## Figures and Tables

**Table 1:** Color of fresh and dried orange slices obtained by FD or HD.

Sample	*L*	*a*	*b*	Δ*E*
Fresh	47.54 ± 0.40 ^c^	−0.60 ± 0.07 ^c^	10.06 ± 0.61 ^c^	−
FD	74.90 ± 0.61 ^a^	4.36 ± 0.38 ^a^	30.47 ± 0.92 ^a^	34.39 ± 0.35 ^a^
HD	49.21 ± 0.65 ^b^	3.35 ± 0.37 ^b^	19.48 ± 0.68 ^b^	10.11 ± 0.19 ^b^

FD: vacuum freeze-drying; HD: hot-air drying; −: not applicable; Different lowercase letters in the same column indicate a significant statistical difference at *p* < 0.05.

**Table 2:** Content of functional ingredients in fresh and dried orange slices obtained by FD or HD.

Sample	VC (g/100 g)	TA (g CA/100 g)	TFC (g QE/100 g)	TPC (g GA/100 g)	TSS (g Glu/100 g)
Fresh	0.22 ± 0.01 ^a^	1.23 ± 0.12 ^b^	1.52 ± 0.09 ^b^	0.70 ± 0.06 ^b^	39.13 ± 2.53 ^a^
FD	0.20 ± 0.02 ^a^	1.51 ± 0.08 ^a^	1.56 ± 0.03 ^b^	0.67 ± 0.03 ^b^	32.35 ± 2.36 ^b^
HD	0.16 ± 0.00 ^b^	1.36 ± 0.02 ^ab^	1.81 ± 0.05 ^a^	0.83 ± 0.03 ^a^	28.56 ± 3.14 ^b^

FD: vacuum freeze-drying; HD: hot-air drying; All data were expressed on a dry matter basis, taking into account the moisture content. VC: vitamin C; TA: titritable acid; TFC: total flavonoid content; TPC: total phenolic content; TSS: total soluble sugars; Different lowercase letters in the same column indicate a significant statistical difference at *p* < 0.05; g CA/100 g: grams of citric acid equivalent per 100 g of sample; g QE/100 g: grams of quercetin equivalent per 100 g of sample; g GA/100 g: grams of gallic acid equivalent per 100 g of sample; g Glu/100 g: grams of glucose equivalent per 100 g of sample.

**Table 3:** Primary flavonoids content and antioxidant capacity of fresh and dried orange slices obtained by FD or HD.

Sample	Narirutin (g/100 g)	Hesperidin (g/100 g)	Didymin (g/100 g)	ABTS (g VC/100 g)	DPPH (g VC/100 g)	FRAP (g VC/100 g)
Fresh	0.46 ± 0.01 ^a^	1.56 ± 0.18 ^b^	0.05 ± 0.00 ^b^	0.70 ± 0.01 ^a^	0.35 ± 0.02 ^a^	0.31 ± 0.00 ^a^
FD	0.34 ± 0.02 ^b^	1.51 ± 0.05 ^b^	0.06 ± 0.01 ^b^	0.62 ± 0.02 ^b^	0.32 ± 0.01 ^b^	0.26 ± 0.01 ^b^
HD	0.47 ± 0.01 ^a^	1.82 ± 0.08 ^a^	0.09 ± 0.01 ^a^	0.57 ± 0.01 ^c^	0.29 ± 0.01 ^c^	0.21 ± 0.00 ^c^

FD: vacuum freeze-drying; HD: hot-air drying; All data were expressed on a dry matter basis, taking into account the moisture content. ABTS: 2,2′-azino-bis(3-ethylbenzothiazoline-6-sulfonic acid); DPPH: 1,1-diphenyl-2-picrylhydrazyl radical; FRAP: ferric ion reducing antioxidant power; VC: vitamin C; Different lowercase letters in the same column indicate a significant statistical difference at *p* < 0.05; g VC/100 g: grams of vitamin C equivalent per 100 g of sample.

## Data Availability

The original contributions presented in this study are included in the article/Supplementary Material. Further inquiries can be directed to the corresponding author.

## Supplementary Materials

The following supporting information can be downloaded at: https://www.mdpi.com/article/10.3390/foods14244327/s1, Figure S1: Proposed pathways for the bioconversion of the monoterpene during the drying process.
