# Supplementary material for: A Comparative Study of Vacuum-Freeze-Dried and Hot-Air-Dried Gannan Navel Orange Slices: Physical Characteristics, Volatile/Non-Volatile Compounds, Antioxidant Activity, and Sensory Attributes
Source: Foods. 2025 Dec 15;14(24):4327. doi: 10.3390/foods14244327 (PMC12732380; doi:10.3390/foods14244327)
Supplement: Supplementary file 1 [file foods-14-04327-s001.zip › foods-4034237-supplementary.pdf]

Supplementary material

A comparative study of vacuum-freeze-dried and hot-air-dried Gannan navel orange slices: physical characteristics, volatile/non-volatile compounds, antioxidant activity, and sensory attributes

Yan Liang [1,a], Qingna Wu [1,a], Qin Xiong [1], Jun Zhang [1,*]

[1] National Engineering Research Center of Navel Orange, Gannan Normal University, Ganzhou, 341000, China; zjhxy110@126.com (Y.L.); wuqingna2022@163.com (Q.W.); skyxiongqin@163.com (Q.X.);

[a] These authors contributed to this work equally

[*] Correspondence: tzhangjun@gnnu.edu.cn

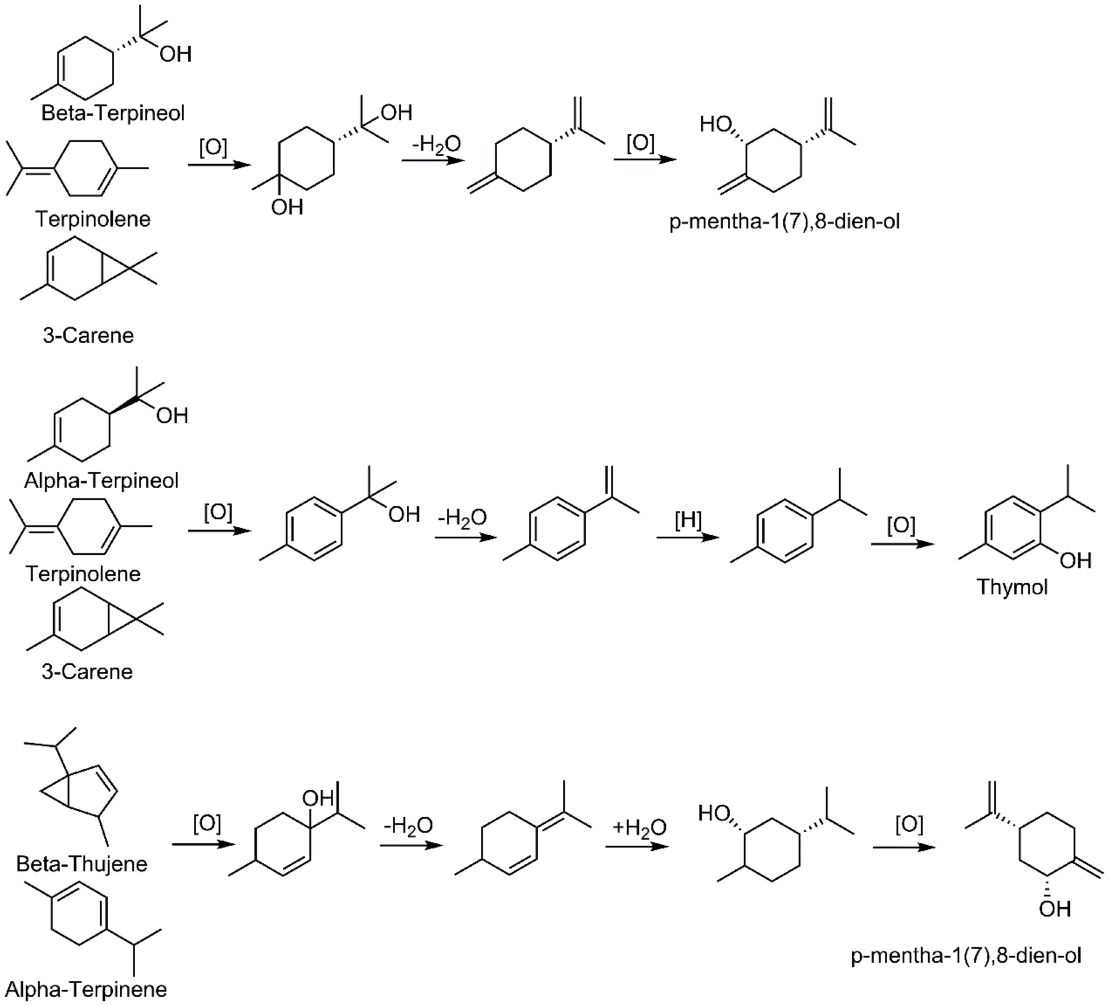

Figure S1. Proposed pathways for the bioconversion of the monoterpene during the drying process.
